# Supplementary material for: Vibrational signature of hydrated protons confined in MXene interlayers
Source: Nat Commun. 2023 Mar 10;14:1322. doi: 10.1038/s41467-023-36842-0 (PMC10006414; doi:10.1038/s41467-023-36842-0)
Supplement: Supplementary file 1 — Supplementary Information [file 41467_2023_36842_MOESM1_ESM.docx]

Supplementary information

Vibrational signature of hydrated protons confined in MXene interlayers

Mailis Lounasvuori^1^, Yangyunli Sun^2^, Tyler S. Mathis^3^, Ljiljana Puskar^1^, Ulrich Schade^1^, De-En Jiang^2†^, Yury Gogotsi^3^, Tristan Petit^1^*

**Affiliations:**

^1^Helmholtz-Zentrum Berlin für Materialien und Energie; Berlin, Germany

^2^Department of Chemistry, University of California, Riverside; Riverside, CA, United States

^3^Department of Materials Science and Engineering and A. J. Drexel Nanomaterials Institute, Drexel University; Philadelphia, PA, United States

^†^Current address: Department of Chemical and Biomolecular Engineering, Vanderbilt University: Nashville, TN, US

*Corresponding author. Email: [tristan.petit@helmholtz-berlin.de](mailto:tristan.petit@helmholtz-berlin.de)


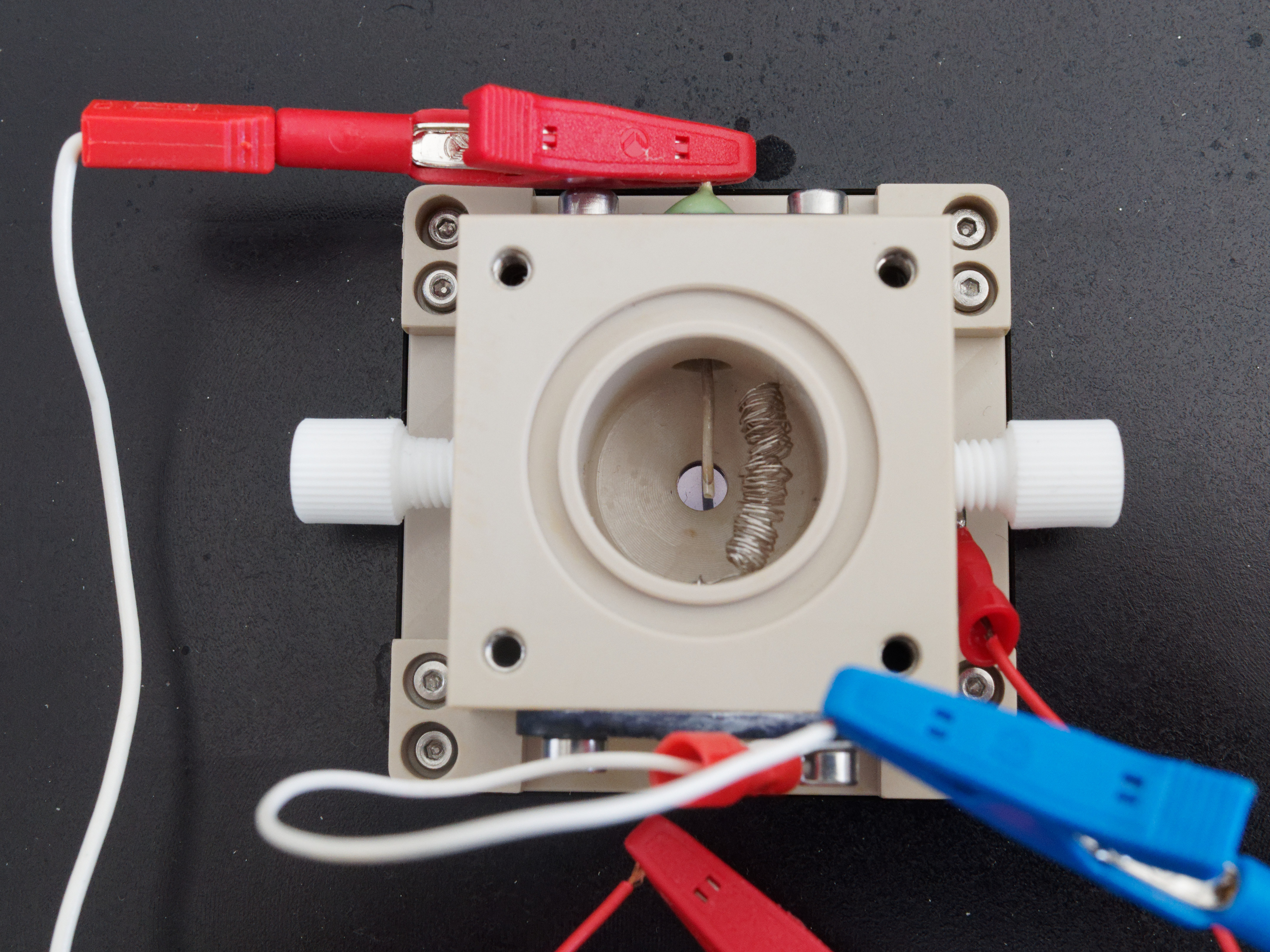


**Supplementary Fig. 1. The spectroelectrochemical cell for *operando* FTIR.** Top view of the spectroelectrochemical cell used in this study.

**Supplementary Note 1: Probing depth.** The thickness of the MXene film was determined with a stylus profiler (Bruker Dektak)*.* Similar FTIR results were obtained with a thinner (430 nm) MXene film. The optical constants of Ti_3_C_2_T*_x_* MXene have not yet been measured experimentally in the mid-infrared range. Theory predicts that the refractive index n of Ti_3_C_2_(OH)_2_ in vacuum is in the range 2.5-4.9 and the extinction coefficient k is as high as 2 (*1*), but the presence of voids in the film as well as intercalated water and ions will hugely impact the optical properties of the film. Based on the theoretical value, the ATR condition is not met using a Si ATR element and 28.74° angle of incidence. Work is ongoing to develop a model that more accurately describes the MXene film in order to calculate the penetration depth and absorbance spectra. In the meantime, we have investigated the penetration depth experimentally by gluing a piece of Scotch tape onto MXene films of different thicknesses (Supplementary Fig. 2). The adhesive in the tape ensures good contact with the underlying substrate and provides strong IR absorbance signal as can be seen when the tape is glued onto a clean Si wafer. When the tape is glued onto an 80-nm-thick MXene film, some absorbance features are visible in the spectrum. When the MXene film thickness reaches about 240 nm, no signal from the tape can be seen anymore. Since the operando measurements described here were performed with films ca. 600 nm thick, we can be sure that the probing depth in these measurements does not extend beyond the MXene film and that we are only probing water present inside the film, either confined between the MXene layers or existing in larger mesopores within the film.

Water within the MXene film may exist confined within MXene sheets but possibly also in larger cavities between stacks of sheets. A spray coated MXene thin film synthesized with a similar mild etching method and delaminated with Li^+^ cations was found to contain macropores and wide mesopores in addition to interlayer spaces, contributing significantly to the water signal detected with EQCM-D (electrochemical quartz crystal microbalance with dissipation monitoring) (*2*). We must therefore acknowledge that the absolute spectra without subtractions would represent both confined and bulk-like water and hydrated protons.


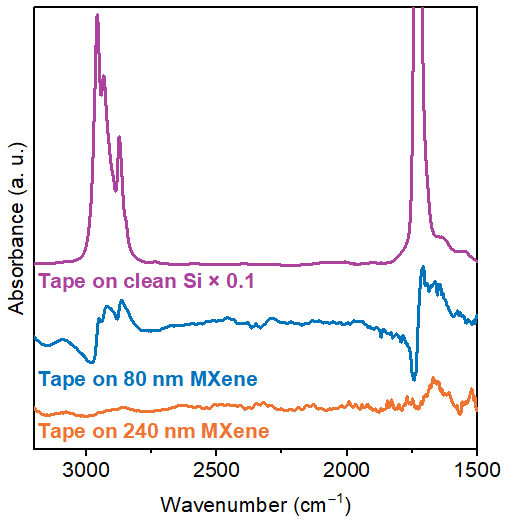


**Supplementary Fig. 2. Probing depth of ATR-FTIR through MXene films.** FTIR spectra of adhesive tape on clean Si and MXene films of different thicknesses. Spectra are offset for clarity.


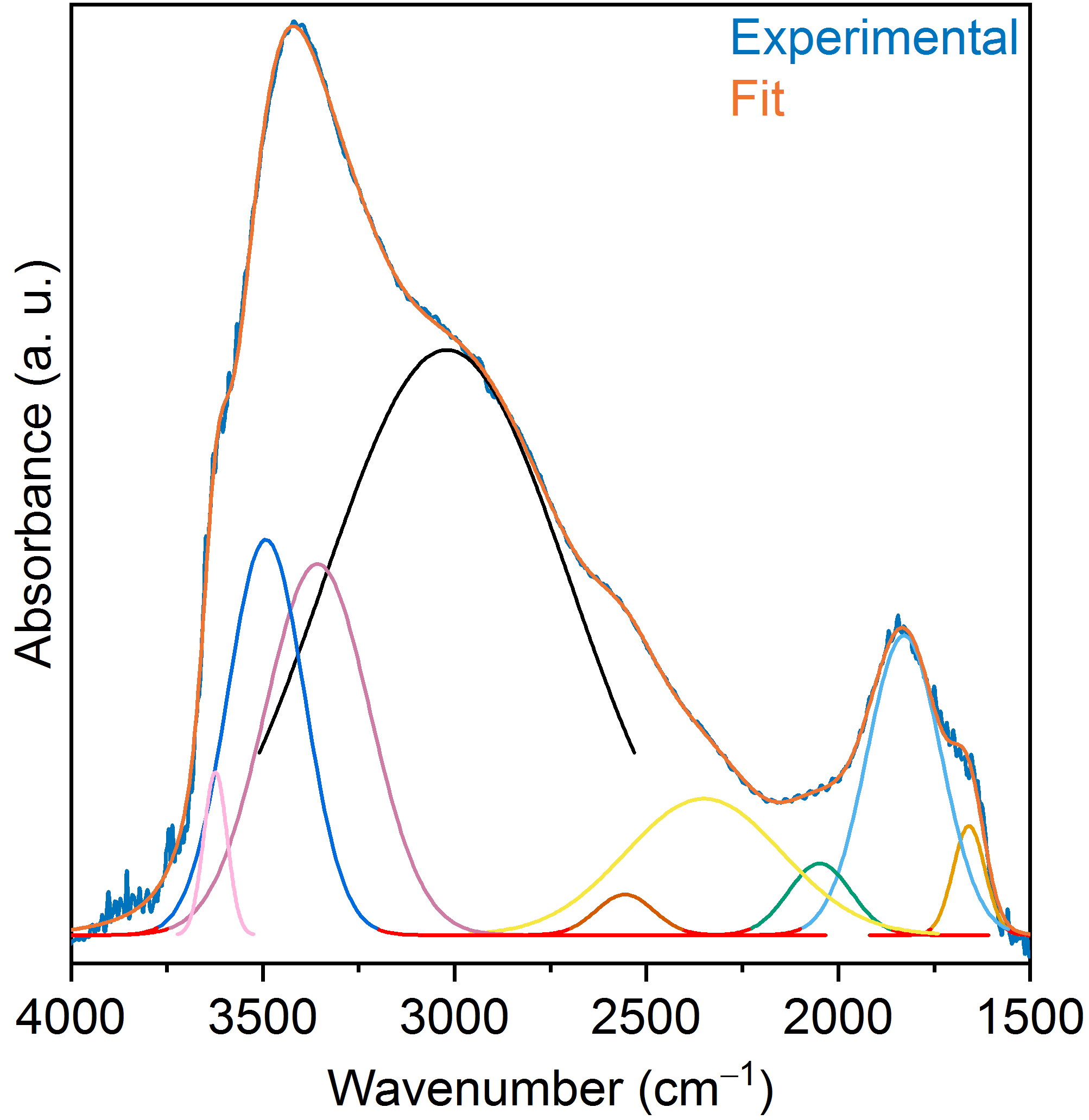


**Supplementary Fig. 3. FTIR spectrum of proton-intercalated Ti_3_C_2_T*_x_* MXene in vacuum.** Peak fit is shown and related peak parameters are detailed in Supplementary Table 1.

**Supplementary Table 1.** Peak fit results of proton-intercalated Ti_3_C_2_T*_x_* MXene in vacuum (Supplementary Fig. 3).

| Frequency (cm^−1^) | Area | Assignment |
| --- | --- | --- |
| 1660 | 0.20 | Water bending mode |
| 1829 | 1.29 | Hydrated protons |
| 2048 | 0.26 | Hydrated protons |
| 2350 | 1.23 | Hydrated protons |
| 2556 | 0.13 | Hydrated protons |
| 3020 | 8.3 | Hydrated protons/strongly H-bonded water |
| 3358 | 2.25 | H-bonded water |
| 3492 | 1.77 | Weakly H-bonded water |
| 3624 | 0.22 | Free O-H |


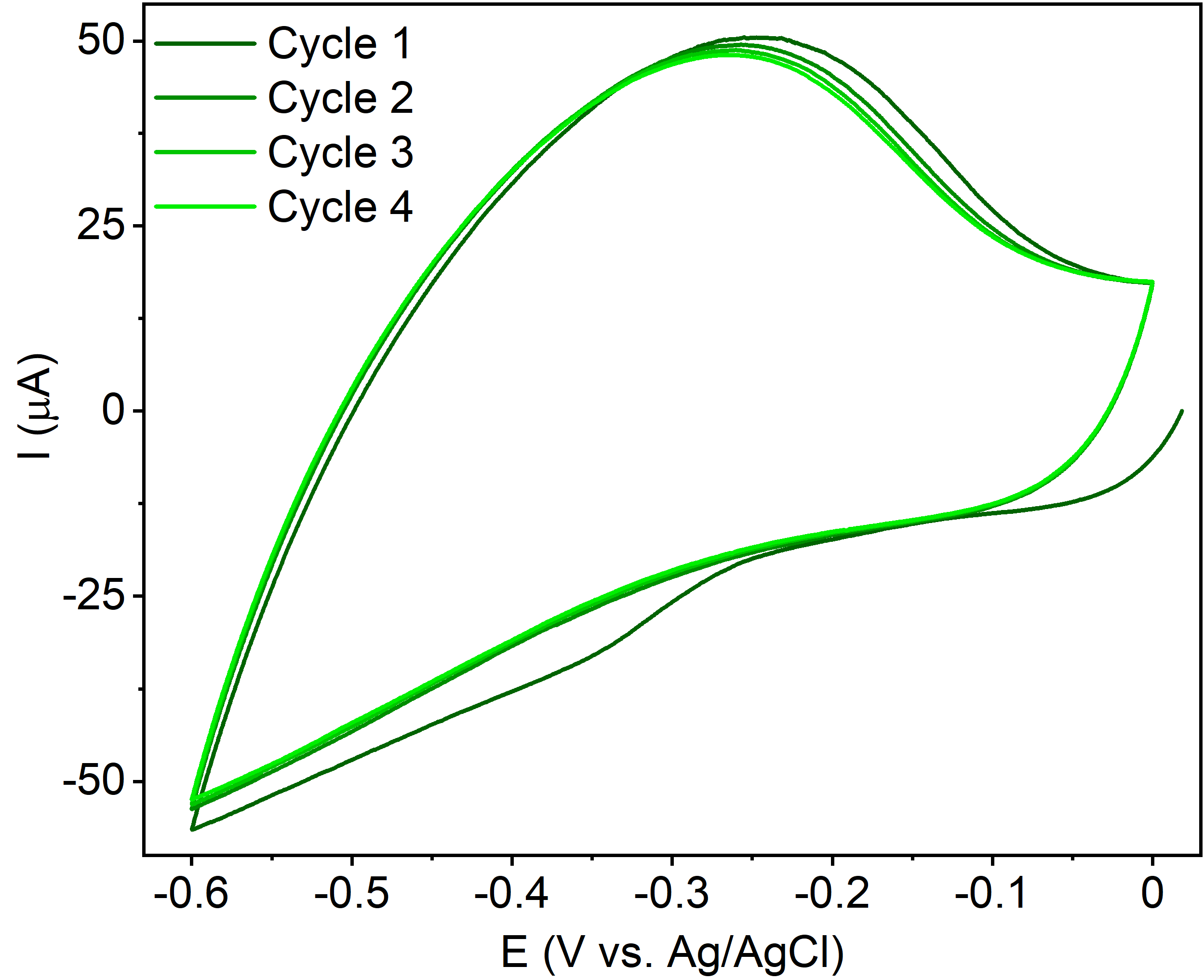


**Supplementary Fig. 4.** **Stability of** Ti_3_C_2_T*_x_* MXene **electrode.** Successive cycles of the cyclic voltammogram recorded in 0.1 M H_2_SO_4_ during the operando FTIR measurements presented in the manuscript.


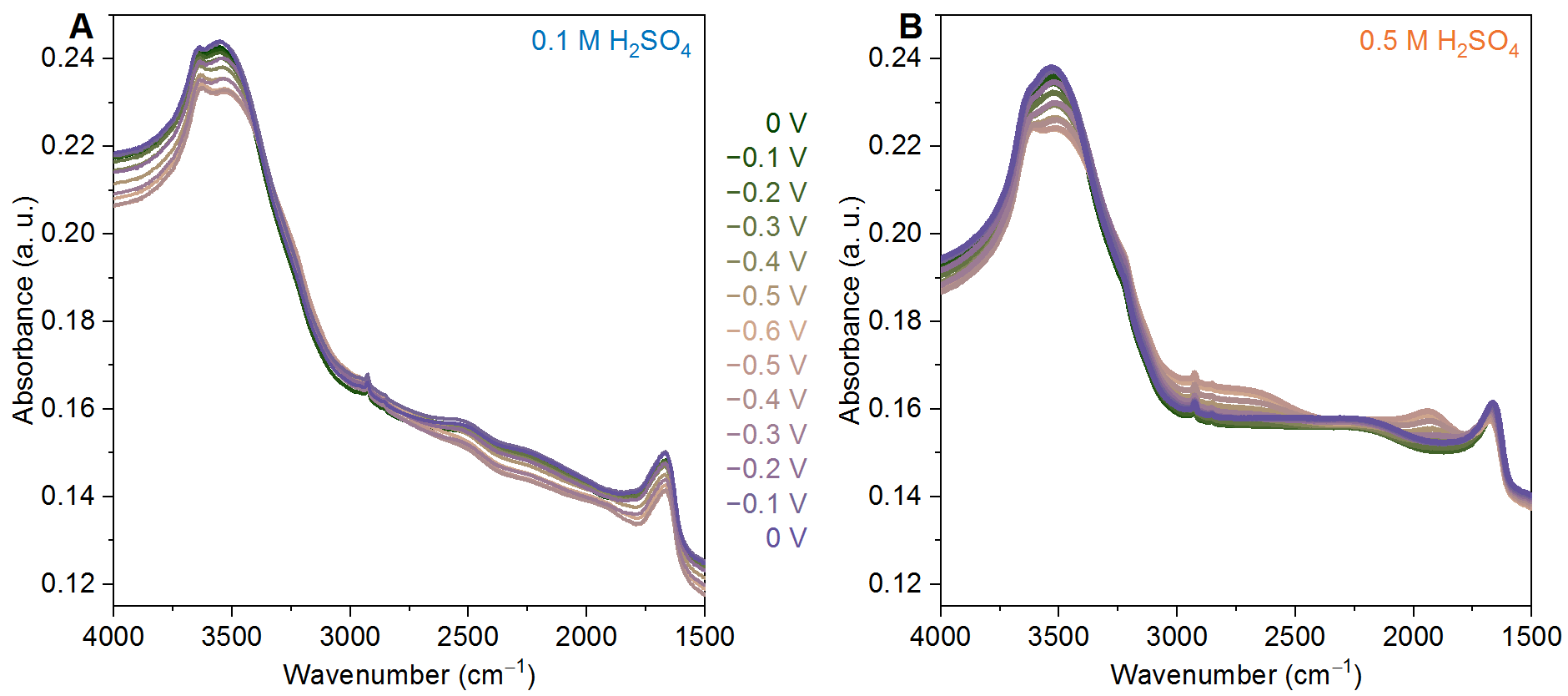


**Supplementary Fig. 5. *Operando* FTIR spectra without subtractions.** *Operando* FTIR spectra of Ti_3_C_2_T*_x_* MXene in 0.1 M (A) and 0.5 M (B) H_2_SO_4_. The spectra are referenced to the graphene-covered Si wafer prior to MXene film deposition.

**Supplementary Note 2: *Operando* FTIR in Li_2_SO_4_ aqueous solution.** Difference spectra recorded in 0.1 M Li_2_SO_4_ electrolyte are manifestly different compared to acidic electrolyte (Supplementary Fig. 6A). No changes appear in the region 3000-1900 cm^−1^, where features from hydrated protons are observed in H_2_SO_4_. No negative bands in the O-H stretching region are visible in the neutral electrolyte. Instead, a large increase in absorbance is seen, spanning 3600-2900 cm^−1^, attributed to water co-intercalating with Li^+^ ions. The peak absorption shifts to lower frequencies with more negative potential, reflecting the changing H-bonding environment of the water as more Li^+^ is introduced. Peak 7, a sharp positive peak at 3625 cm^−1^, is present in neutral electrolyte as well as in acidic electrolyte. This peak is assigned to dangling O-H bonds of water molecules at the MXene film interface that are unable to participate in the fully H-bonded water network due to confinement. Comparison of the peak fitting in 0.1 M Li_2_SO_4_ and 0.1 M H_2_SO_4_ (Supplementary Fig. 6B, C) reveals that peak 7 appears at almost exactly the same frequency in both electrolytes, but the area is somewhat higher in acidic electrolyte. Peak 7 may therefore include some contribution from -OH surface groups of MXene that are protonated at more negative potentials. CVs recorded during *operando* IR measurements are shown in Supplementary Fig. 5D.


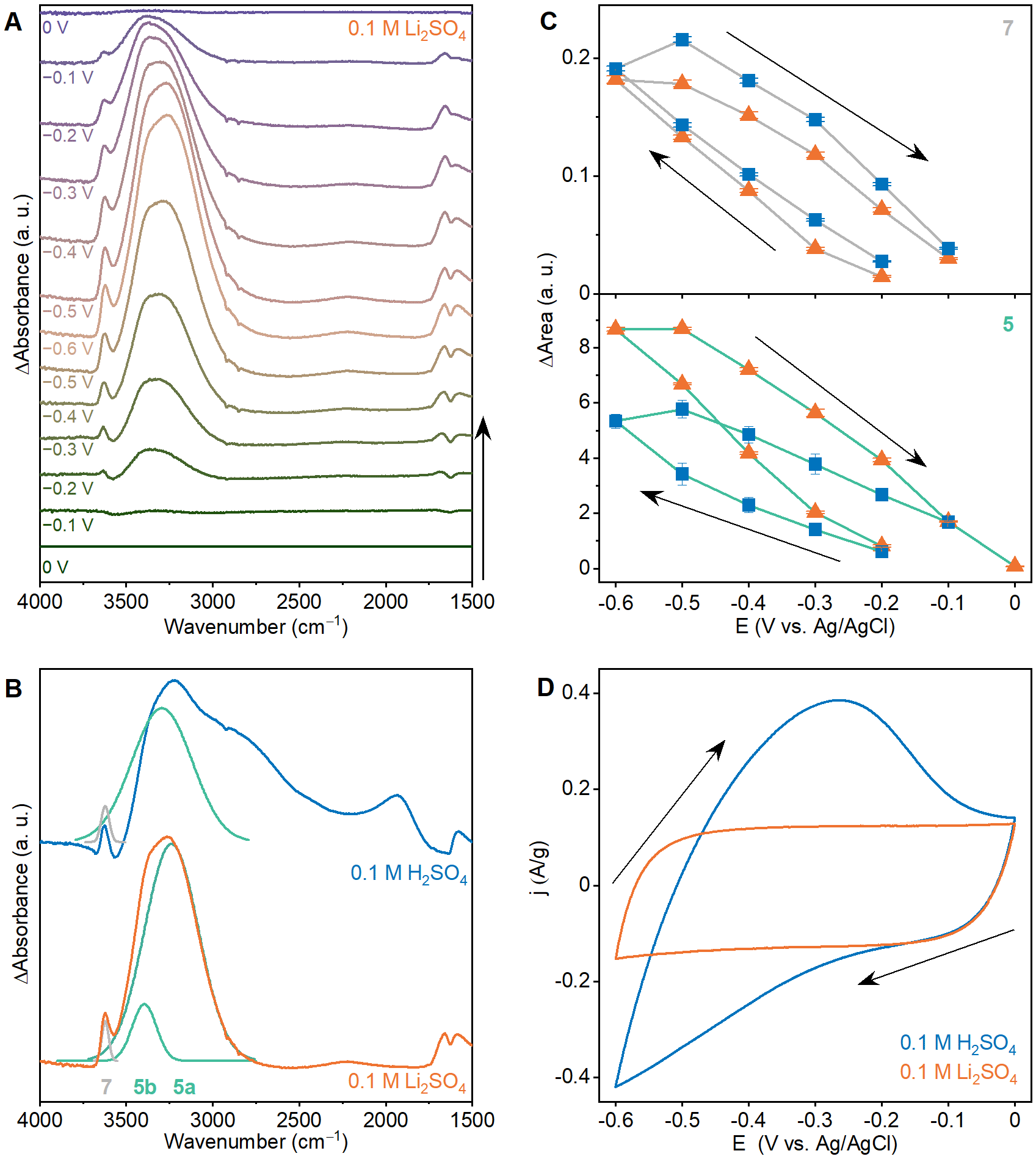


**Supplementary Fig. 6.** **Comparison of acidic electrolyte with neutral electrolyte.** (A) *Operando* FTIR difference spectra of Ti_3_C_2_T*_x_* MXene in neutral 0.1 M Li_2_SO_4_ electrolyte during cyclic voltammetry. The spectrum at 0 mV was subtracted from subsequent spectra. Spectra are offset for clarity. (B) Peaks 5 and 7 fitted to difference spectra in acidic and neutral electrolyte recorded at −0.6 V. In neutral electrolyte, peak 5 is represented by two components a and b. (C) Areas of peaks 5 and 7 plotted as a function of potential with error bars representing the standard deviation. Blue square symbols: acidic electrolyte; orange triangle symbols: neutral electrolyte. In neutral electrolyte, the area of peak 5 is the sum of components a and b. (D) CVs recorded during *operando* FTIR measurement.

**Supplementary Note 3: Deuterated spectra.** To support the assignment of spectral features to hydrated protons, operando FTIR spectra were recorded in 0.1 M D_2_SO_4_ in D_2_O (Supplementary Fig. 7). The wavenumber axis has been scaled by the ratio of the D_2_O and H_2_O gas phase antisymmetric stretches *(3)*. Upon deuteration we see matching features related to deuterons that are shifted to lower frequencies (2000 cm^−1^ and 1130 cm^−1^). Overall, the wavenumber region covering peaks 1-4 is consistent with measurements in H_2_SO_4_, whereas the wavenumber region covering the water-related bands 5-7 shows diverging behavior in deuterated electrolyte. These differences in the higher frequency tail of the O-H/O-D stretch feature warrant further examination which is currently ongoing.


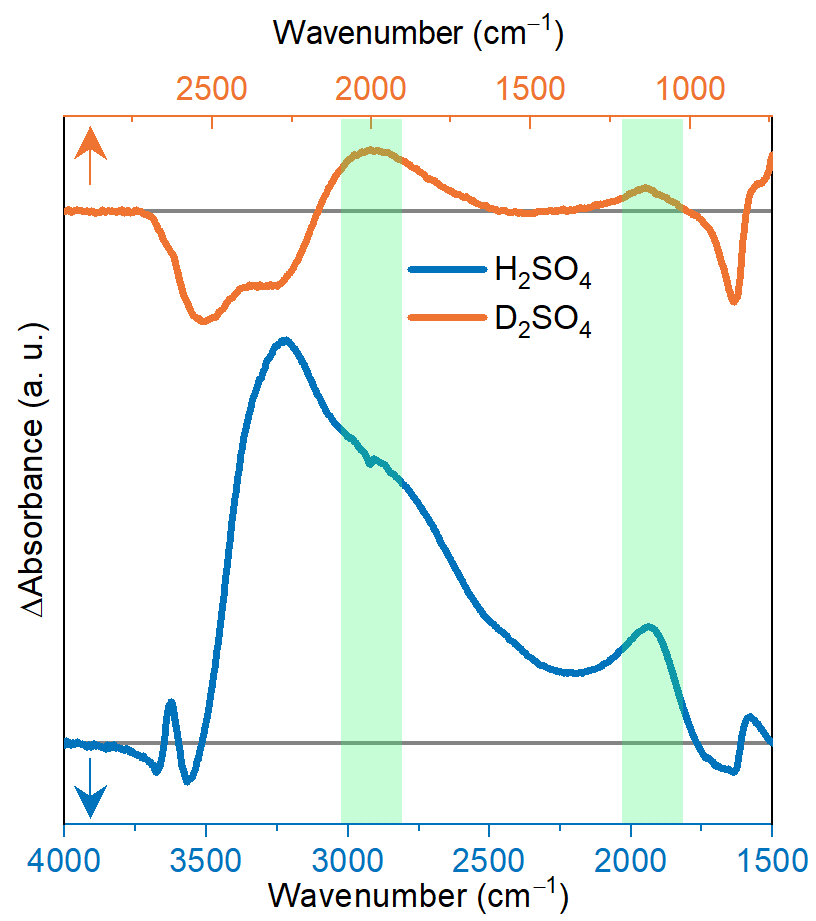


**Supplementary Fig. 7. Comparison of light and deuterated electrolytes.** *Operando* FTIR difference spectra of Ti_3_C_2_T*_x_* MXene in 0.1 M H_2_SO_4_ and 0.1 M D_2_SO_4_ recorded at −0.6 V and the spectrum at 0 V subtracted. The green shaded areas highlight the matching features in the spectra, assigned to confined hydrated protons/deuterons.


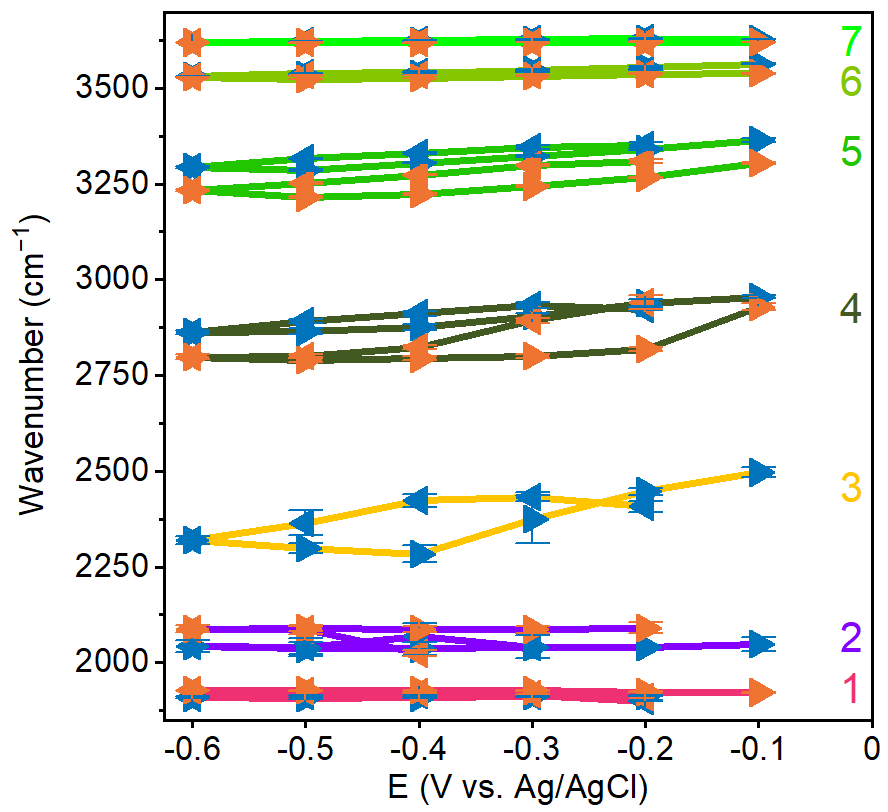


**Supplementary Fig. 8.** **Frequencies of peak fitting components.** Peak frequencies extracted from peak fitting of the *operando* FTIR data in 0.1 M H_2_SO_4_ (blue symbols) and 0.5 M H_2_SO_4_ (orange symbols). Error bars represent the standard deviation.

**Supplementary Note 4: Estimation of intercalated proton concentration in the MXene electrodes.** The MXene thin film was formed of approx. 125 μg of Ti_3_C_2_T*_x_*. The electrode area was defined by an O-ring with inner diameter of 7 mm. Assuming the formula Ti_3_C_2_O_2_, we have 6.81×10^−7^ mol formula units in the Ti_3_C_2_T*_x_* thin film electrode.

0.1 M H_2_SO_4_: The negative and positive charge passed during one cycle in the *in situ* measurement were −12.4 and +11.9 mC. Assuming all charge is associated with protons intercalating into the MXene film, we have 0.19 protons per Ti_3_C_2_T*_x_* formula unit (1.29×10^−7^ mol) intercalating into the MXene film and 0.18 protons per formula unit (1.23×10^−7^ mol) deintercalating out of the film.

0.5 M H_2_SO_4_: The negative and positive charge passed during one cycle in the *in situ* measurement were −14.5 and +14.6 mC. Assuming all charge is associated with protons intercalating into the MXene film, we have 0.22 protons per Ti_3_C_2_T*_x_* formula unit (1.51×10^−7^ mol) intercalating into the MXene film and 0.22 protons per formula unit (1.52×10^−7^ mol) deintercalating out of the film.

Some of the film was outside of the inner diameter of the O-ring and therefore not in contact with the electrolyte.

**Supplementary Table 2.** Scaled frequencies for all normal modes contributing to the peak at ca. 1900 cm^−1^ in the calculated spectrum with one proton confined between Ti_3_C_2_T*_x_* MXene layers.

| **Frequency (cm^−1^)** | **δ (Å)** | $\boldsymbol{r}_{\boldsymbol{O}_{\boldsymbol{d}}\boldsymbol{H}}$ **(Å)** | $\boldsymbol{r}_{\boldsymbol{O}_{\boldsymbol{a}}\boldsymbol{H}}$ **(Å)** | **Relative intensity** | **Corresponding species** |
| --- | --- | --- | --- | --- | --- |
| 1912 | 0.23 | 1.08 | 1.32 | 7.52 | Hydronium core + special pair water |
| 1922 | 0.15 | 1.10 | 1.25 | 6.42 | Hydronium core + special pair water |
| 1798 | 0.32 | 1.08 | 1.40 | 5.53 | Hydronium core +1^st^ solvation shell water |
| 1953 | 0.25 | 1.09 | 1.34 | 5.42 | Hydronium core + special pair water |
| 1873 | 0.29 | 1.11 | 1.40 | 2.02 | Hydronium core |

**Supplementary Table 3.** Scaled frequencies for all normal modes contributing to the peak at ca. 2200 cm^−1^ in the calculated spectrum with one proton confined between Ti_3_C_2_T*_x_* MXene layers.

| **Frequency (cm^−1^)** | **δ (Å)** | $\boldsymbol{r}_{\boldsymbol{O}_{\boldsymbol{d}}\boldsymbol{H}}$ **(Å)** | $\boldsymbol{r}_{\boldsymbol{O}_{\boldsymbol{a}}\boldsymbol{H}}$ **(Å)** | **Relative intensity** | **Corresponding species** |
| --- | --- | --- | --- | --- | --- |
| 2252 | 0.36 | 1.07 | 1.43 | 6.14 | Hydronium core + special pair water |
| 2260 | 0.15 | 1.08 | 1.23 | 5.26 | Hydronium core + special pair water |
| 2102 | 0.40 | 1.08 | 1.48 | 4.85 | Hydronium core + special pair water |
| 2253 | / | 1.08 | / | 3.76 | Special pair water |
| 2200 | 0.48 | 1.08 | 1.56 | 2.54 | Hydronium core |
| 2139 | 0.42 | 1.08 | 1.50 | 2.53 | Hydronium core |
| 2201 | 0.49 | 1.10 | 1.59 | 2.46 | Hydronium core |
| 2200 | 0.30 | 1.09 | 1.39 | 1.11 | Hydronium core |

**Supplementary Table 4.** Scaled frequencies for all normal modes contributing to the peak at ca. 2100 cm^−1^ in the calculated spectrum with three protons confined between Ti_3_C_2_T*_x_* MXene layers.

| **Frequency (cm^−1^)** | **δ (Å)** | $\boldsymbol{r}_{\boldsymbol{O}_{\boldsymbol{d}}\boldsymbol{H}}$ **(Å)** | $\boldsymbol{r}_{\boldsymbol{O}_{\boldsymbol{a}}\boldsymbol{H}}$ **(Å)** | **Relative intensity** | **Corresponding species** |
| --- | --- | --- | --- | --- | --- |
| 1994 | 0.43 | 1.06 | 1.49 | 13.08 | Hydronium core |
| 2081 | 0.43 | 1.09 | 1.52 | 12.92 | Hydronium core |
| 2146 | / | 1.07 |  | 10.38 | Surface hydroxyl group |
| 2266 | 0.47 | 1.01 | 1.47 | 8.82 | Hydronium core |
| 2018 | 0.44 | 1.07 | 1.51 | 8.19 | Hydronium core |
| 2099 | 0.16 | 1.08 | 1.24 | 7.59 | Hydronium core |
| 2104 | 0.22 | 1.07 | 1.29 | 7.12 | Hydronium core |
| 2184 | 0.35 | 1.07 | 1.42 | 6.71 | Hydronium core + water |
| 2061 | 0.36 | 1.10 | 1.45 | 6.49 | Hydronium core |
| 2210 | 0.01 | 1.17 | 1.18 | 6.38 | Hydronium core |
| 2050 | 0.29 | 1.08 | 1.37 | 5.93 | Hydronium core |
| 2122 | 0.12 | 1.15 | 1.27 | 5.85 | Hydronium core |
| 2187 | 0.37 | 1.07 | 1.44 | 3.99 | Hydronium core |
| 1981 | 0.56 | 1.05 | 1.61 | 3.82 | Hydronium core |
| 2119 | 0.34 | 1.03 | 1.38 | 3.52 | Hydronium core + special pair water |
| 2225 | 0.39 | 1.06 | 1.45 | 3.31 | Hydronium core |
| 2166 | 0.23 | 1.18 | 1.41 | 3.15 | Zundel |
| 2076 | / |  |  | 1.72 | Hydronium core + water |

**
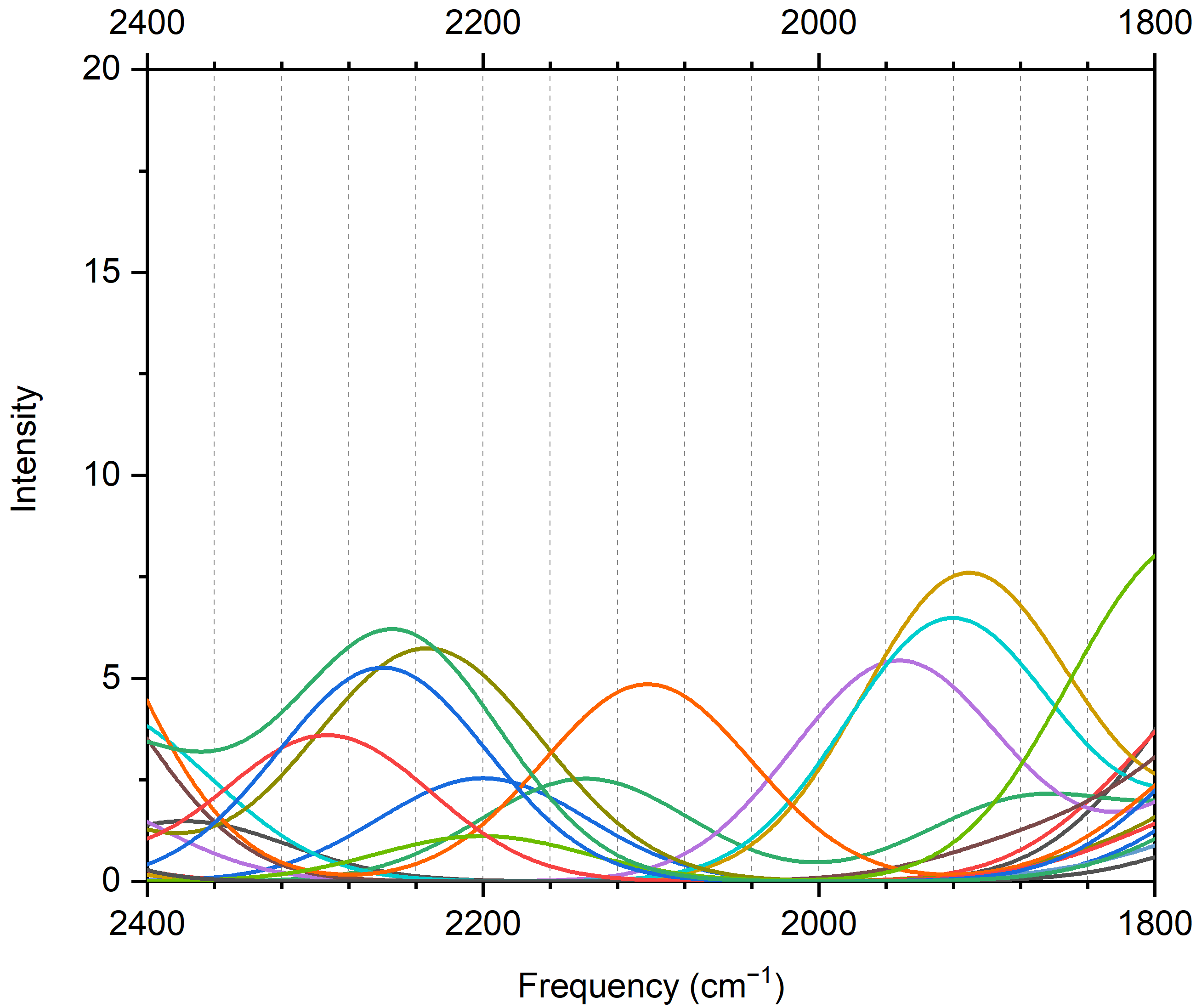
**

**Supplementary Fig. 9. Comparison of simulated spectra.** Simulated IR spectra for 16 snapshots from the AIMD simulation of a typical proton concentration (Ti_3_C_2_O_2_·1.42H_2_O·0.08H); each line represents one snapshot.


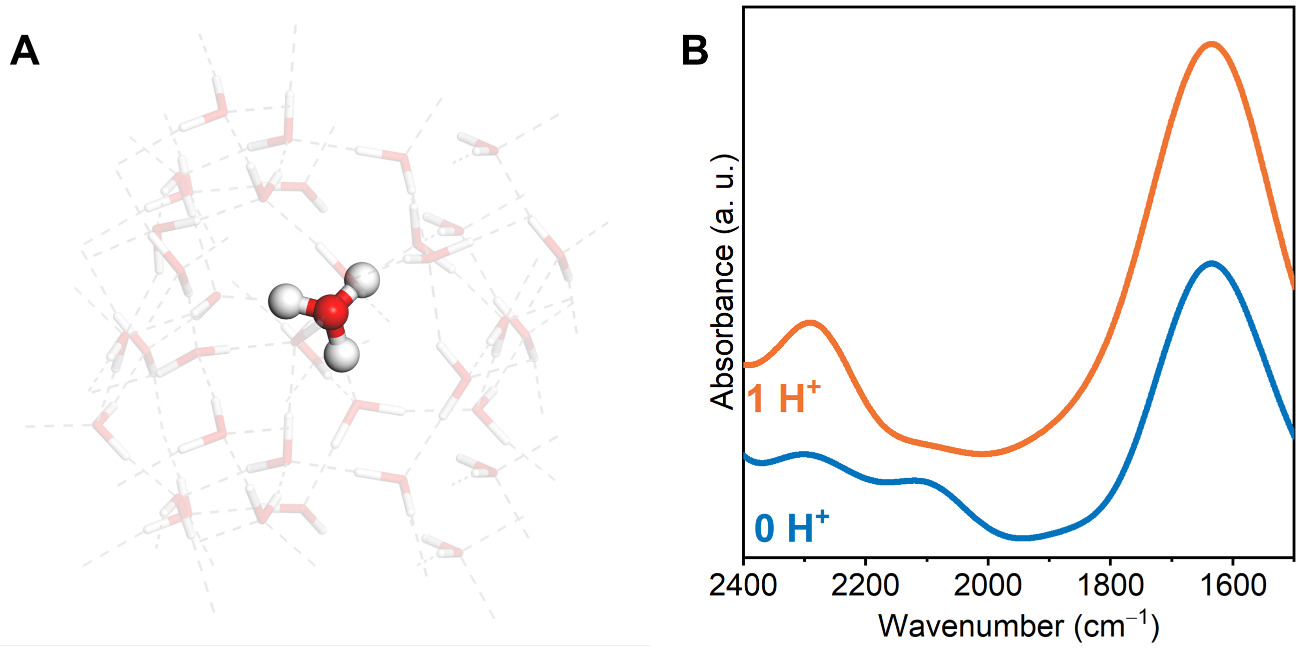


**Supplementary Fig. 10. DFT calculations for bulk water.** (A) Structure from calculation with one proton in bulk water; (B) simulated spectra with 0 and 1 protons in bulk water.

**Supplementary References**

1. G. R. Berdiyorov, Optical properties of functionalized Ti_3_C_2_T_2_ (T = F, O, OH) MXene: First-principles calculations. *AIP Advances* **6**, 55105 (2016). DOI: 10.1063/1.4948799
2. N. Shpigel, M. D. Levi, S. Sigalov, T. S. Mathis, Y. Gogotsi, D. Aurbach, Direct assessment of nanoconfined water in 2D Ti_3_C_2_ electrode interspaces by a surface acoustic technique. *J. Am. Chem. Soc*. **140**, 8910–8917 (2018). DOI: 10.1021/jacs.8b04862
3. L. De Marco, W. Carpenter, H. Liu, R. Biswas, J. M. Bowman, A. Tokmakoff, Differences in the vibrational dynamics of H_2_O and D_2_O: observation of symmetric and antisymmetric stretching vibrations in heavy water. *J. Phys. Chem. Lett.* **7** (10), 1769–1774 (2016). DOI: 10.1021/acs.jpclett.6b00668
